# A Bill to Insure the Better Education of Practitioners of Dental Surgery Etc.

**Published:** 1886-03

**Authors:** 


### A Bill,

### To Insure the Better Education of Practitioners of Dental Surgery and to Regulate the Practice of Dentistry in the State of Ohio.

*Be it enacted by the General Assembly of the State of Ohio:*

SECTION 1. It shall be unlawful for any person who is not at the time of the passage of this Act engaged in the practice of dentistry in this State, in conformity to the laws of Ohio existing at the time of the enactment hereof, to commence such practice, unless such person shall have obtained a certificate as hereinafter provided.

SEC. 2. A Board of Examiners, to consist of five practicing dentists, is hereby created, whose duty it shall be to carry out the purposes and enforce the provisions of this Act. The members of said Board shall be appointed by the Governor, who shall select them from ten candidates, whose names shall be furnished him by the Ohio State Dental Society. The term for which the said members of said Board shall hold their offices shall be three years, except that two of the members of the Board, first to be appointed under this Act, shall hold their office for the term of one year, two for the term of two years, and one for the term of three years, respectively, and until their successors shall be duly appointed and qualified. The Governor shall determine by lot the terms of the first appointees of this Board. In case of a vacancy occurring in said Board, such vacancy shall be filled by the Governor in conformity with this section.

SEC. 3. Said Board shall organize immediately after its appointment by choosing one of its members President and one the Secretary thereof, and it shall meet at least once in every year and as much oftener and at such time and place as it may deem necessary. A majority of said Board shall at all times constitute a quorum, and the proceedings thereof shall, at all reasonable times, be open to public inspection.

SEC. 4. Within six months from the time that this Act takes effect, it shall be the duty of every person who is now lawfully engaged in the practice of dentistry in this State, to cause his or her name, place of business and residence to be registered with said Board of Examiners, who shall keep a book for that purpose. The statement of every such person shall be verified under oath before a Notary Public, in such manner as may be prescribed by the Board of Examiners. Every person who shall so register with said Board as a practitioner of dentistry shall receive a certificate to that effect, and may continue to practice as such without incurring any of the liabilities or penalties provided in this Act, and shall pay to the Board of Examiners for such registration the sum of one dollar. It shall be the duty of the Board of Examiners to forward to the County Recorder of each county in the State a certified list of all persons residing in his county who have registered in accordance with the provisions of this Act, and it shall be the duty of County Recorders to register such names in a book, to be kept for that purpose.

Sec. 5. Any person, who shall so desire, may appear before said Board at any of its regular meetings and be examined with reference to his or her knowledge and skill in dental surgery, and said Board shall issue to such person as it shall find to possess the requisite qualifications a certificate to that effect, in accordance with the provisions of this Act. Said Board shall also indorse as satifactory, diplomas from any reputable dental college, upon the holder furnishing evidence satisfactory to the Board of his or her right to the same, and shall issue certificates to that effect within ten days thereafter. All certificates issued by said Board shall be signed by its officers, and such certificates shall be prima facie evidence of the right of the holder to practice dentistry in the State of Ohio.

Sec. 6. Any person who shall violate any of the provisions of this Act shall be deemed guilty of a misdemeanor, and, upon conviction, may be fined not less than fifty dollars nor more than two hundred dollars, and shall be committed to the county jail until such fine be paid, or confined in the county jail for each and every offense. And it shall be incumbent on the defendant to show that he has authority under the law to practice dentistry to exempt himself from the penalty hereby prescribed. All fines recovered under this Act shall be paid into the Common School Fund of the county in which said conviction takes place.

Sec. 7. In order to provide the means for carrying out and maintaining the provisions of this Act, the said Board of Examiners shall charge each person applying for a certificate and having a diploma from a reputable dental college a fee of five dollars and each other person appearing before them for examination for a certificate of qualification a fee of ten dollars, which fee shall in no case be returned, and out of the funds coming into the possession of the Board from the fees so charged, and penalties received under the provisions of this Act, all legitimate and necessary expenses incurred in attending the meetings of said Board shall be paid. And no part of the expenses of the Board shall ever be paid out of the State Treasury. All moneys received in excess of expenses, above provided for, shall be held by the Secretary of said Board as a special fund for meeting the expenses of said Board, and carrying out the provisions of this Act, he giving such bonds as the Board shall from time to time direct. And said Board shall make an annual report of its proceedings to the Governor, by the first of January of each year, together with an account of all moneys received and disbursed by them pursuant to this Act.

Sec. 8. Any person who shall receive a certificate from said Board to practice dentistry, shall cause his or her certificate to be registered with the County Recorder of the County in which such person may reside, and the County Recorder shall charge for registering such certificate a fee of one dollar. Any failure, neglect, or refusal on the part of any person holding such certificate to register the same with the County Recorder as above directed, for a period of three months, shall work a forfeiture of the certificate; and no certificate when once forfeited shall be restored, except upon the payment to the Board of Examiners of the sum of twenty-five dollars, as a penalty for such neglect, failure, or refusal.

Sec. 9. Any one who shall knowingly or falsely claim or pretend to have a certificate or license, diploma, or degree granted by the Board of Examiners, under and pursuant to the provisions of this Act, or who shall falsely and with intent to deceive the public, claim or pretend to be a graduate from any incorporated dental college, shall be deemed guilty of a misdemeanor, and shall be liable to the same penalty as provided in section six.

Sec. 10. Any member of the Board of Examiners may grant a permit to practice until the next meeting of the Board, but such permit shall be valid only until said next meeting, and shall in no case be extended except as provided in section five of this Act.

SEC. 11. Nothing in this Act shall be so construed as to prohibit any practicing physician or surgeon from extracting teeth.

SEC. 12. That original sections 4404 and 6991 be and the same are hereby repealed; and this Act shall take effect and be in force from and after its passage.

